# Books and Authors

**Published:** 1909-05

**Authors:** 


### BOOKS AND AUTHORS.

**International Clinics.** A Quarterly of Illustrated Clinical Lectures and especially prepared articles on Treatment, Medicine, Surgery, Neurology, Pediatrics, Obstetrics, Gynecology, Orthopedics, Pathology, Dermatology, Ophthalmology, Otology, Rhinology, Laryngology, Hygiene and other topics of interest to students and practitioners. Edited by **W. T. Longcope,** M.D., Volume I. Nineteenth series. Philadelphia and London: J. B. Lippincott Co. 1908. (Cloth, $2.00.)

These volumes appear four times a year with such regularity, and have continued to do so for eighteen years, that their popularity is attested by these facts. In this book there are two articles on treatment; four on medicine; four on surgery; two on gynecology and obstetrics; one on genitourinary disease; one on proctology; one on rhinology; one on dermatology; and one on pathology. Besides these there is a review of the progress of medicine for 1908, that part upon treatment being prepared by A. A. Stevens; that relating to medicine, by David L. Edsall, and Verner Nisbet; and that pertaining to surgery by Joseph C. Bloodgood. This addition to the regular articles serves to make this volume one of unusual interest, the resume on progress being exceedingly well excerpted. The clinical articles proper are in general presented by men of experience, some of whom are very well known in their respective fields of labor. The book is of interest and value, complimenting its predecessors in excellent fashion.

**Progressive Medicine.** A Quarterly Digest of Advances, Discoveries and Improvements in the Medical and Surgical Sciences. Edited by **Hobart Amory Hare,** M.D., Professor of Therapeutics and Materia Medica in the Jefferson Medical College of Philadelphia, Vol. XI, No. 1, March, 1909, Octavo, 277 pages. Per annum, in four paper-bound volumes, containing 1,200 pages. Lea & Febiger, Publishers, Philadelphia and New York: ($6.00, net; in cloth, $9.90, net.)

In the issue of this periodical before us we find surgery of the head, neck and thorax, occupying about one hundred pages, prepared by Charles H. Frazier; infectious diseases, including acute rheumatism, influenza, and croupous pneumonia, excerpted by Robert B. Preble and covering over fifty pages; diseases of children, occupying thirty-two pages, and made up by Floyd M. Crandall; rhinology and laryngology, dealt with by D. Braden Kyle; and otology handled by Arthur B. Duel. Dr. Crandall directs attention to Dr. F. Park Lewis's (Buffalo) recommendation relating to the prevention of ophthalmia neonatorum. Dr. Lewis insists that midwives as well as physicians shall instill a drop of a 1 per cent. silver nitrate solution in the eyes of every new born infant. This dictum cannot be presented too often; as only by "continually hammering" can it be made clear that 30 per cent. of blindness can thereby be prevented. The book is full of original material offered in an interesting and instructive form.

---

**Orthopedic Surgery for Practitioners.** By **Henry Ling Taylor,** M.D. Professor of Orthopedic Surgery and Attending Orthopedic Surgeon, New York Post-Graduate Medical School and Hospital, etc.; assisted by **Charles Ogilvy,** M.D., Adjunct Professor of Orthopedic Surgery at the same institution; and by **Fred H. Albee,** M.D., Instructor of Orthopedic Surgery at the same institution. With 254 illustrations. New York and London: D. Appleton & Company. 1909. (Price, $5.00, cloth.)

This work is prepared by an expert orthopedic surgeon not, however, for the specialist, but for the general practising physician. It is well that this should be so, for he rather than the specialist generally sees deformed children and even adults before they go to the orthopedist. He even has the opportunity of treating the deforming diseases in their early stages and before the resultant deformities have become fixed or permanently distorted. It is during these earlier manifestations that frequently simpler methods, comparatively speaking, prove effectual.

The author attempts to place in the hands of any intelligent practitioner such methods of treatment as will result in success, putting aside useless or indifferent material. He makes three divisions of his book—namely, general, special, and technical, the several parts being essential to a complete presentation of the subject in its most scientific form. The general part deals with causes, congenital crippling affections, nutritional disorders, infections, diseases of unknown origin, tumors and cysts, malignant diseases, spontaneous fracture, ununited fracture, diseases of the nervous system, examination and diagnosis, prevention, prognosis, treatment of underlying cause, complications, and treatment of deformity. The latter includes bandaging, strapping, splinting and all mechanical apparatus; also operative treatment, exercises and gymnastics.

In the special part the author handles all deformities and deals with them in sections—first, in the neck and trunk, next in the shoulder girdle and upper extremity, and finally in the pelvic girdle and lower extremity. In the last part technic is considered in all its details, such as means of increasing and diminishing local pressure, increasing and restricting motion, bandaging, strapping, splinting, general and special, material such as plaster of paris, celluloid, leather, steel and the like, leg splints,—ankle, knee and hip—pelvic, arm, spinal and neck splints. In fact, nothing is neglected that science and art, working together, can devise or apply for the cure of deformity, congenital or acquired.

Taylor's twenty-five years' experience, surrounded as he has been by men of great skill and scientific attainments in orthopedics, qualifies him to express opinions and deal with questions of great difficulty in an understanding manner, that imparts clearness to the reader, as well as satisfaction to the learner.

The book is beautifully printed on heavy calendered paper and the illustrations portray the author's meaning and intent with emphasis. A list of orthopedic literature and an excellent index add a finish to the work, that is one of the best treatises on the subject.

---

**Transactions of the American Surgical Association.** Vol. XXVI. Edited by **Richard H. Harte,** Recorder. Printed for the Association by William J. Dornan, Philadelphia. 1908.

Although this great association has put forth many excellent volumes since it was organised by Samuel D. Gross in 1880, it is doubtful if any have been as resplendent as this one. All the papers are of exceptional good quality while many of them are brilliant. Mr. Moynihan, of Leeds, accentuates his second visit to the association by reading a paper entitled "Late results after operations for benign diseases of the stomach and duodenum," it being the first of a series of discourses on stomach and duodenal diseases, with special reference to their surgical cure. The other participants in this symposium were William J. Mayo, John B. Deaver, William L. Rodman, John B. Roberts and F. E. Bunts. It was a notable company and the contributions were worthy the men comprising it. John B. Murphy read a sterling paper on perforative peritonitis, that was followed by a brilliant discussion, during the course of which Mr. Moynihan, while taking part, paid high tribute to Dr. Murphy.

We should like to deal, in extenso, with this splendid volume but inasmuch as it is available for those who may wish to possess it, we hardly think it desirable to do so, particularly as we've said quite enough to give a hint of its value to any one who practises surgery. It is a beautifully printed, well indexed book of seven hundred and fifty-six pages.

---

**Transactions of the Section on Obstetrics and Diseases of Women of the American Medical Association** at the 59th annual session held at Chicago, June 2, 5, 1908.

This section at its last meeting was presided over by Dr. Walter Blackburn Dorsett, of St. Louis. He chose for the subject of his address, "Criminal Abortion in its Broadest Sense." It is an important topic and was well handled by Dr. Dorsett. The book is filled with excellent papers, some of which are by distinguished men. August Martin, of Berlin, presented the topic of "Genital Tuberculosis," an important topic well set forth and, discussed with ability. The grouping of the work of this section into one volume where each and every contribution can be seen at a glance and studied at one's pleasure is a striking argument against leaving them to the mercies of a journal, where they are seen by few and fewer still read them.

**Parcimony in Nutrition,** by **Sir James Crichton-Browne,** M.D.., LL.D., F.R.S. 12 mo. Funk & Wagnalls Company, New York. (Cloth, 75 cents, net.)

The author of this monograph is so widely known that attention will be attracted to his little work from that fact alone: but over and beyond this, it deals with a topic full of interest to every intelligent man and woman, not to mention physicians themselves. The author made this the subject of his presidential address to the section on preventive medicine at the meeting of the Royal Institute of Public Health at Buxton, England, in July, 1908, and has expanded it for publication in the present form. Sir James regards nutrition as an important branch of preventive medicine. This sentence contains a vast amount of common sense: "Could we but secure adequate supplies and an equitable redistribution of food, restricting gluttony on the one hand and abolishing starvation on the other, we should vastly lighten the task of the sanitary and of the social reformer, and witness a gratifying drop in the death rate." In the next paragraph he says "What must I eat to be saved?" is a question often heard and one " to which answer is made by a shouting multitude of enthusiasts, cranks, and empirics, each with an infallible dietetic system of his own." Sir James attacks Professor Chittenden's investigations and statements in the most courteous manner, nevertheless forcefully and convincingly. He admits that the distinguished Yale professor has dealt with the question of the appropriate proteid intake "in a manner that is at once painstaking and brilliant, laborious and fascinating."

Briefly stated Chittenden's conclusion is "that the daily amount of proteid or albuminous food required for the maintenance of health and vigor is not more than one-half that hitherto regarded as necessary." Sir James enters the classics describing food and meals in Homeric times, and speaks of the culture of the Greeks and their liberal supply of meat food. The book is most charmingly presented in choice English and is highly instructive of the topic chosen, which itself is unique.

**Transactions of the Fourteenth Annual Meeting of the American Laryngological, Rhinological, and Otological Society,** held at Pittsburg, May 28, 29, and 30, 1908. Saint Louis: Published by the Society. 1908.

The organisation of this society is somewhat unusual. Besides one general body, there are four geographical sections. Eastern, Middle, Southern, and Western. Meetings are held in each section once a year at periods of time that do not conflict.

This volume contains the proceedings of the annual meeting held at Pittsburg, May 28, 30, 1908, and of the four section meetings. It is filled with scientific papers of interest, some of which are elaborately illustrated. The book is well printed and substantially bound. The society is a credit to the department of medicine with which it deals.

---

**Report of the Commissioner of Education of the United States** for the year ended June 30, 1907. Vols. I-II. By **Elmer Ellsworth Brown,** Ph.D., LL.D. Washington: Government Printing Office. 1908.

This interesting report contains much of value to educators. The bureau, established in 1867 as a department, was created an office in the Interior Department in 1869. Its growth has been slow, not commensurate with the increase and development of education in the United States, but under the five several commissioners that have presided over it, a substantial foundation has been laid. It is interesting to learn from this report that Congress appropriates $100,000 for educational purposes in Alaska, with which fifty-two public day schools have been conducted for natives, with an enrollment of 2,639, and an average attendance of 1,139. It is a curious fact that the Alaska division not only has charge of schools, but also of the government reindeer in that territory, now numbering over 15.000 head. They are mostly owned by the government, but Eskimos and Laplanders, and also the mission societies of several churches are owners of smaller herds.

